# Broadband and Tunable RCS Reduction using High-order Reflections and Salisbury-type Absorption Mechanisms

**DOI:** 10.1038/s41598-019-45501-8

**Published:** 2019-06-21

**Authors:** Jiakun Song, Xiaoyu Wu, Cheng Huang, Jianing Yang, Chen Ji, Changlei Zhang, Xiangang Luo

**Affiliations:** 1 State Key Laboratory of Optical Technologies on Nano-Fabrication and Micro-Engineering, Institute of Optics and Electronics, Chinese Academy of Sciences, Chengdu, 610209 China; 2 University of Chinese Academy of Sciences, Beijing, 100049 China

**Keywords:** Electrical and electronic engineering, Applied physics

## Abstract

In this paper, a broadband and tunable radar cross section (RCS) reduction structure is proposed by using the hybrid physical mechanism that is based on high-order reflections and Salisbury-type absorption. Our design combines the high-index grating structure with a traditional Salisbury screen in which the lossy sheet is made of a graphene structure. When it is illuminated by a plane wave with normal incidence, the Salisbury screen can absorb the incoming wave, and the introducing high-index grating structure could further reduce the backward scattering wave by generating high-order reflection beams, which broadens the RCS reduction bandwidth. In addition, the RCS reduction level can be dynamically controlled by tuning the surface resistance of the graphene layer. Simulated results show that the proposed structure can realize tunable RCS reduction between 4.1 and 18 GHz under normal incidence with different graphene resistances. Experimental results are in accordance with those of the simulation results. In addition, the scattering field distributions and the plots of surface power loss density have been illustrated to analyze the RCS-reduction mechanism of our structure.

## Introduction

Radar cross section (RCS) reduction techniques have been extensively investigated in the past decades due to its particularly important applications in civil and military areas. So far, there have been mainly two techniques to control the radar scattering signal. One technique is to adopt the radar absorbing materials to convert the incident radar wave into heat or other kind of energy. Many radar absorbers, such as Salisbury screen^1^, Jaumann absorber^2^, resistive frequency selective surface (FSS)^3,4^ and magnetic absorbing materials^5,6^, have been reported to realize the RCS reduction by absorbing the incident wave. The Salisbury screen is very simple in structure, which is only made of a continuous resistive sheet placed at a quarter-wavelength above the conducting plate^7^. However, its absorption bandwidth is usually very narrow. The Jaumann absorber can improve the absorption bandwidth by employing additional resistive sheets and spacers, but at cost of increasing the total thickness^8^. In order to simultaneously realize broadband absorption and retain a small thickness, the resistive FSS was proposed to reach this goal by appropriately introducing conductive and resistive pattern on one substrate backed by the ground plane^3^. In addition, there have been also many metamaterial absorbers for wideband RCS reduction^9,10^. The other RCS reduction technique is to guide the reflected wave to other directions that do not point to the radar receivers by using the shaped geometries, thus the backward scattering signal can be sharply reduced^11^. In reality, the loss of the scattering signal at this case could be considered as one kind of radiative loss^12^. The recent metasurface has caused much attention due to its strong phase modulation capability, which can control the electromagnetic scattering shape of the objects with nearly no influence on the geometric properties^13–20^. Through modulating reflection phase distribution with proper arrangement of all the meta-atoms in a space/size/shape-variant manner, the backscatter waves could be steered to pre-designed directions. Using this method, we can obtain the predesigned virtual EM shape of the real objects without optimizing its physical shape^21^. So far, most of the current RCS reduction materials or structures only adopted one of the above two techniques to suppress the backward scattering wave. In addition, they remain passive structures with fixed RCS reduction performance.

Recently, some tunable RCS reduction methods have been proposed to dynamically manipulate the reflected wave. For example, by introducing tunable material^22,23^ or active lumped components^24,25^ into the traditional radar absorbers, the absorbing efficiency or frequency can be dynamically controlled. Graphene, as a kind of two-dimensional material, has been also used to construct the tunable radar absorber. In ref.^26^, voltage-controlled graphene was proposed to switch the absorbing efficiency at microwave region by changing its effective sheet resistance. More recently, several approaches have been proposed to extend the tuning frequency band of the microwave reflectivity. However, most of graphene-based tunable absorbers were just numerically investigated without experimental verification^27–29^.

In this paper, we propose a novel broadband and tunable RCS reduction method by simultaneously introducing absorptive loss and radiative loss in the design. The traditional Salisbury screen and high-index dielectric grating structure are integrated into one structure. The graphene layer is used as a lossy sheet of Salisbury screen, which can absorb the incoming wave and then produce the absorption loss. The employed high-index grating structure could generate high-order reflection beams, resulting in the radiative loss. Experimental results show that the 10 dB RCS reduction is realized between 4.4 and 18 GHz, and by applying DC bias voltage on the graphene layer, the RCS reduction level can be further dynamically tuned. The hybrid physical mechanisms are explained by examining the scattering field distributions and surface power loss density.

## Results

### Structure design and its simulation results

**Figure 1:**
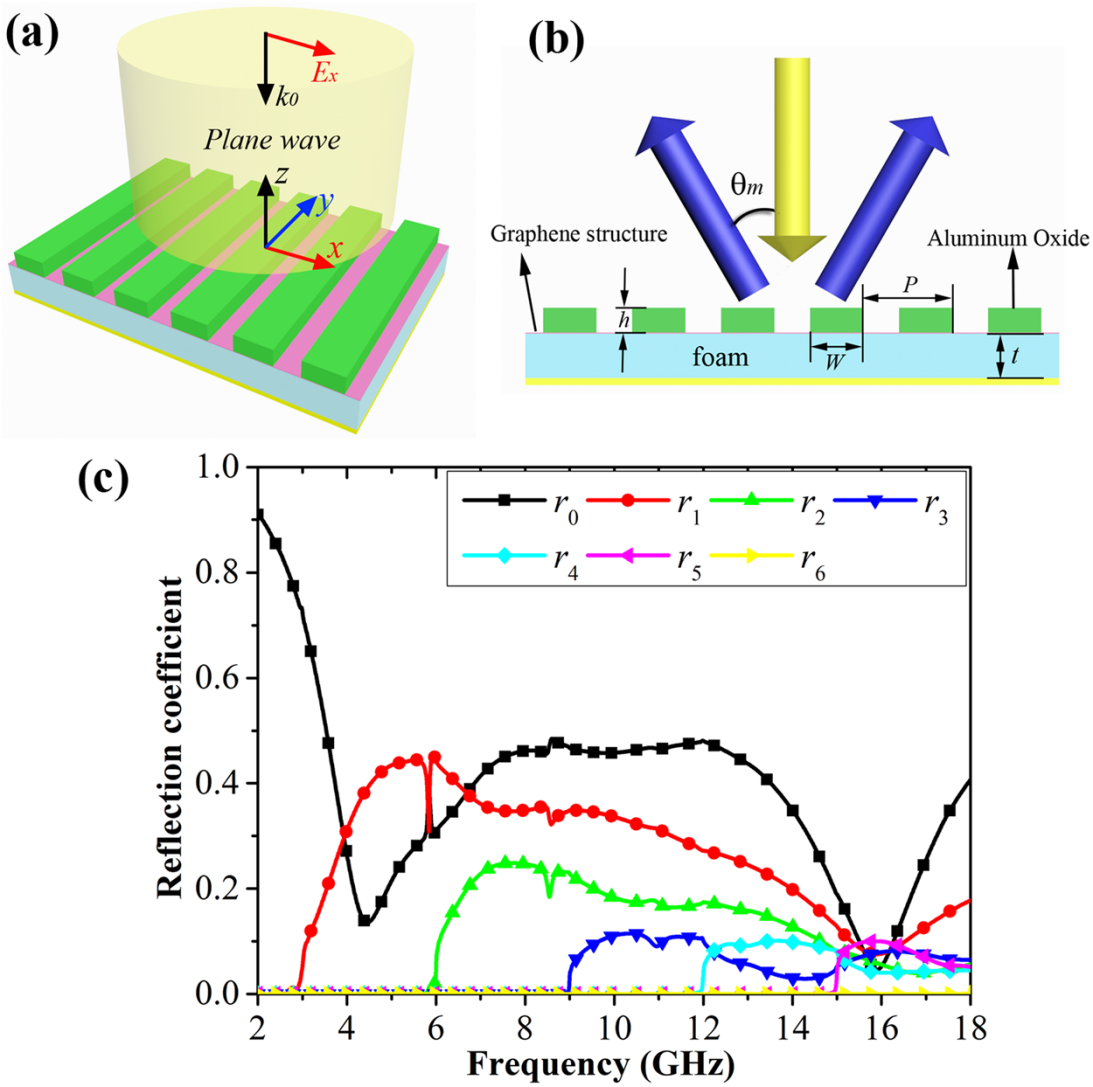
Schematic of the proposed RCS reduction structure and its simulated reflection coefficients. (**a**) Three-dimensional illustration of the 1D grating integrated Salisbury screen. (**b**) Side-view. (**c**) The corresponding reflection coefficients of different reflection orders. The geometrical parameters are optimized as follows: *w* = 65 mm, *h* = 3 mm, *p* = 100 mm, and *t* = 5 mm.

Figure 1(a) shows the proposed RCS reduction structure. It is composed of one-dimensional (1D) high-index dielectric grating structure and the traditional Salisbury screen. The corresponding side view of our structure is given in Fig. 1(b). The 1D high-index grating structure is made of aluminum oxide material (ε = 9.8) and its period is *p* = 100 mm. The width and height of the grating strip are set as *w* = 65 mm and *h* = 3 mm, respectively. The graphene structure is used as a lossy sheet of Salisbury screen, which is attached to a 5 mm-thick foam slab (ε = 1.1) backed with a metal plate. In this design, the grating period is set to be larger than the incident wavelength, so our structure illuminated by an x-polarized plane wave with normal incidence will generate a series of high-order reflection beams, and their reflection angles can be derived from the grating equation *Psinθ*_*m*_ = *mλ*. Here, *P*, *θ*_*m*_ and *λ* are the grating period, the *m*th order reflection angle and incident wavelength, respectively. It is found that all the high-order reflection beams will be deflected from the normal at this case, and then the backward scattering wave is reduced as desired. In addition, the use of the Salisbury screen can further absorb the incoming wave for suppressing the backward scattering wave and realize RCS reduction over a relatively wide frequency band. Numerical simulation is carried out to investigate the reflection characteristics of our structure by using the commercial software CST Microwave Studio. Figure 1(c) shows the simulated reflection coefficients of the high-order reflection beams in our structure under normal incidence. Here, we use *r*_0_, *r*_1_, *r*_2_, *r*_3_, *r*_4_, *r*_5_, and *r*_6_ to represent the reflection coefficients of the different reflection orders, respectively. The surface resistance of the graphene layer on Salisbury screen is first set as *R*_*s*_ = 500 Ω/sq. With the modulation of the 1D high-index grating structure, the strong 1st and 2nd order reflection are generated over a wide frequency band, while other higher-order reflections are relatively very weak. The 1st and 2nd order reflection intensity is quickly enhanced at first and then gradually decreases with the increase of frequency, reaching the minimum around 16 GHz. For the 0th order reflection beam, there are two obvious reflection dips around 4.5 and 16 GHz. The enhancement of the high order diffraction beams results in the backward scattering reduction of the 0th order diffraction beam. Besides, the resistive loss of the Salisbury screen also contributes to the formation of the reflection dip.

**Figure 2:**
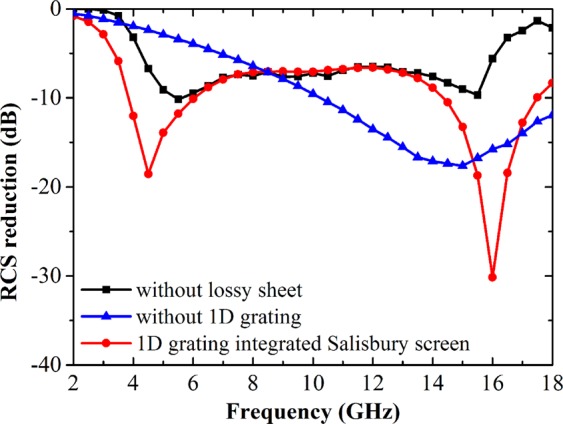
Simulated RCS reduction performance of the designed 1D grating integrated Salisbury screen.

Figure 2 shows the simulated RCS reduction performance. Here, the 1D grating structure with the four periodic cells is adopted in the full-wave simulation. It is seen that the bare Salisbury screen only produces one RCS reduction dip around 15 GHz, while there is no obvious RCS reduction dip for the 1D grating structure in the whole frequency band of interest. However, after integrating 1D grating structure with Salisbury screen, the two RCS reduction dips are respectively generated at 4.5 GHz and 16 GHz where the RCS reduction intensities are also sharply improved.

**Figure 3:**
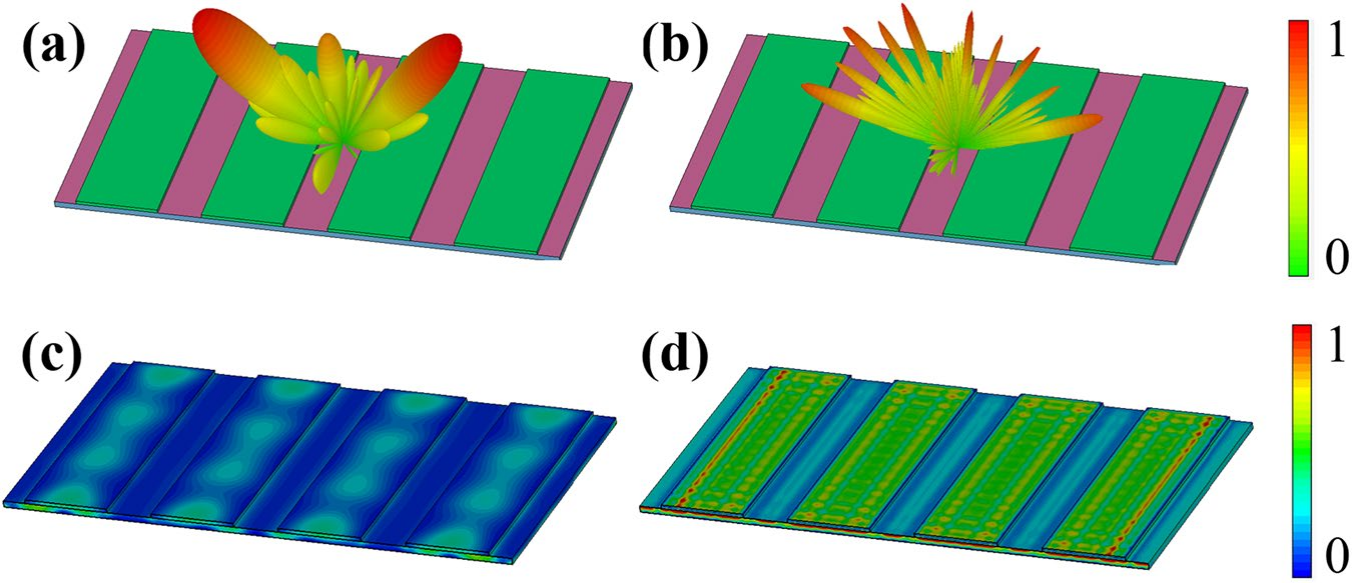
Full-wave simulation results of the proposed 1D grating integrated Salisbury screen under normal incidence of EM waves. The 3D scattering pattern at (**a**) 4.5 GHz and (**b**) 16 GHz. The power loss density distributions at (**c**) 4.5 GHz and (**d**) 16 GHz.

To further understand the physical mechanism of the designed RCS reduction structure, its scattering patterns at 4.5 GHz and 16 GHz are numerically investigated, as displayed in Fig. 3(a,b), respectively. It is seen in Fig. 3(a) that there are three reflection orders (0, 0), (−1, 0) and (1, 0) produced at 4.5 GHz. The main lobes corresponding to the (−1, 0) and (1, 0) reflection orders, are respectively redirected to (−40°, 0) and (40°, 0), which complies with the theoretically calculated results from the grating equation. At the frequency of 16 GHz, a series of high orders reflection beams are generated and redirected to various directions due to the grating period far larger than the operating wavelength. However, the whole high-order reflection efficiency is relatively weak compared with that at 4.5 GHz. The power loss density distributions of our structure are also examined, as seen in Fig. 3(c,d). It is obvious that very strong power loss is focused on the lossy sheet layer at 16 GHz, while the power loss is relatively weak at 4.5 GHz. Therefore, we can deduce that the Salisbury screen plays the leading role in the backward scattering reduction at 16 GHz by absorbing the most of the incident wave energy. The main reason for the generation of the RCS reduction dip at 4.5 GHz is due to the employed high-index grating structure which can generate the strong 1st order reflection around 4.5 GHz, as seen in Fig. 1(c). In addition, the lossy graphene layer can also absorb part of the incident wave energy, as seen in Fig. 3(c). So we consider that the RCS reduction dip at 4.5 GHz originates from the combination of the generation of high-order reflection and the absorption from the lossy graphene layer.

**Figure 4:**
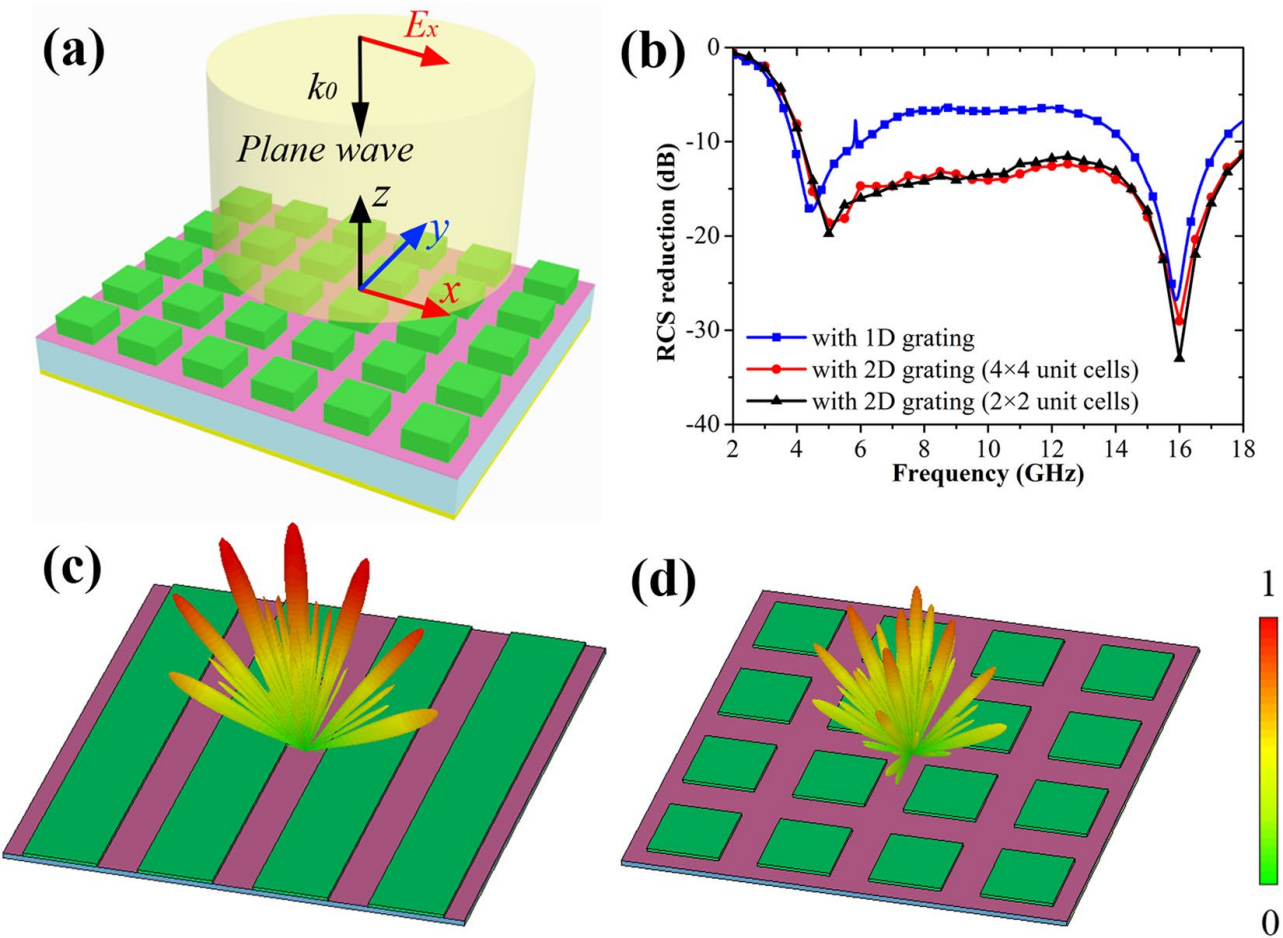
Schematic of the proposed 2D grating integrated Salisbury screen and its RCS reduction performance. (**a**) 3D-view of the 2D grating integrated Salisbury screen. (**b**) Simulated RCS reduction performance of the grating integrated Salisbury screen under normal incidence. (**c**,**d**) Simulated 3D scattering patterns of the proposed 1D and 2D grating integrated Salisbury screen at 10 GHz.

It is worthwhile to point out that between the two RCS reduction dips, there is still a broad band in which the RCS reduction level is less than 10 dB for the 1D grating integrated Salisbury screen. In order to solve this issue, we use the two-dimensional (2D) grating structure instead of the 1D grating structure in our design, as seen in Fig. 4(a). The period along *x* direction *P*_*x*_ is equal to the period along *y* direction *P*_*y*_. Therefore, the electromagnetic response would be polarization independent due to its symmetry structure. Figure 4(b) shows the corresponding RCS reduction performance of the designed 2D grating integrated Salisbury screen with 4 × 4 periodic cells under normal incidence. It is seen that its 10 dB RCS reduction bandwidth is obviously increased, which is expanded from 4.1 to 18 GHz. It may be due to that the 2D grating structure has higher diffraction efficiency and can generate high order reflection in both *yoz* and *xoz* planes, compared with the 1D grating structure. In addition, we still investigate the RCS reduction performance of the proposed structure with only 2 × 2 unit cells. It has been verified to perform almost the same RCS reduction performance with that of 4 × 4 unit cells, as seen in Fig. 4(b). That is to say, the minimum requirement for the grating cells is only two periods in our design. Figure 4(c) shows the 3D scattering pattern of the 1D grating structure. It is seen that the high-order reflection beams are only generated in *xoz* plane, and then the backward reflected wave is still strong. After employing the 2D grating structure, the high-order reflection beams can be simultaneously produced in both *xoz* and *yoz* plane, so the normal reflection of the incoming wave can be further suppressed, as seen in Fig. 4(d).

**Figure 5:**
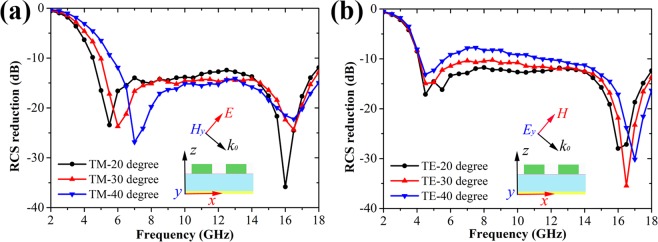
Simulated bistatic RCS reduction performance of the designed 2D grating integrated Salisbury screen with 2 × 2 unit cells under different oblique incident angles. (**a**) TM polarization. (**b**) TE polarization.

**Figure 6:**
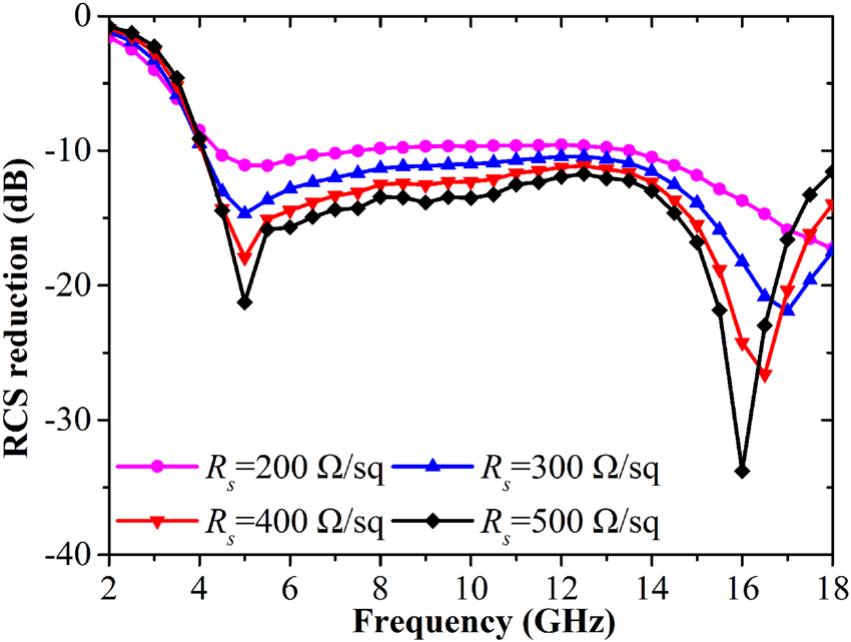
Simulated RCS reduction performance of the designed 2D grating integrated Salisbury screen with the different graphene resistances.

Figure 5 displays the simulated RCS reduction performance under oblique incidence. It is seen that the RCS reduction bandwidth decreases a little under TM mode with the increase of the incident angle. Similarly, the RCS reduction level is also slightly degraded under TE mode. However, low RCS properties are still kept for the proposed 2D grating integrated Salisbury screen. As the effective sheet resistance of graphene can be tuned through an applied electrostatic field bias^26^, the tunable RCS reduction performance could be expected for our structure. As Fig. 6 shows, the proposed structure under normal incidence can dynamically control the RCS reduction level by tuning the graphene resistance from 500 Ω/sq to 200 Ω/sq. The original two RCS reduction dips occurring at 4.5 and 16 GHz disappear at the case of *R*_*s*_ = 200 Ω/sq. That means the graphene resistance of the Salisbury screen has great influence on the RCS reduction level.

### Experimental results

**Figure 7:**
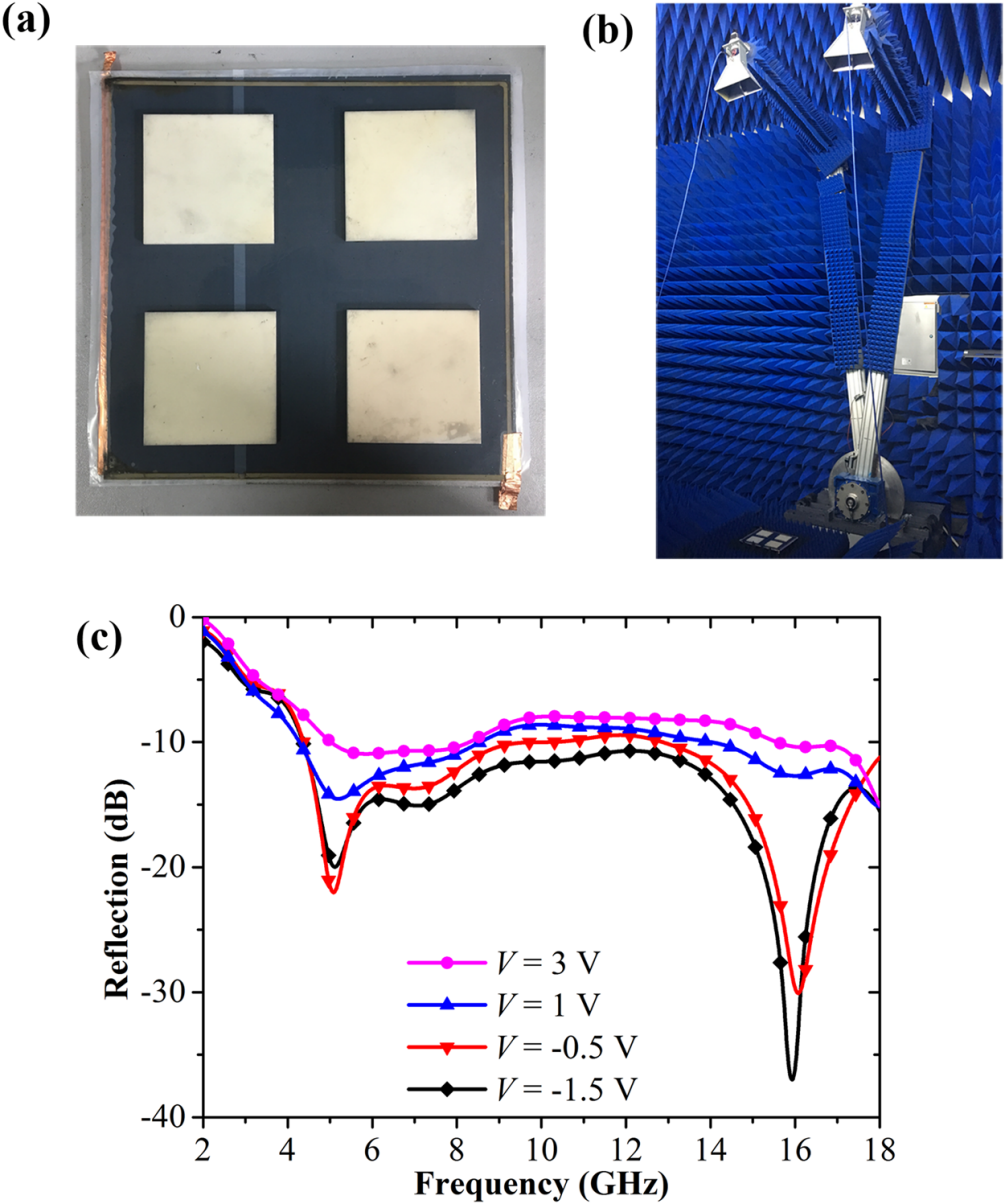
Photograph of measurement setup for the fabricated sample and its corresponding measurement results. (**a**) Photograph of the fabricated sample. (**b**) Photograph of the arch measurement system. (**c**) Measured reflection characteristics of the fabricated sample under normal incidence with the different DC biasing voltages applied on the graphene layer.

In order to verify the broadband and tunable RCS reduction property, we fabricated a design sample composed of 2 × 2 unit cells, as seen in Fig. 7(a). The graphene film is grown on copper foil using the chemical vapor deposition method and then transferred onto the PET film. The thin silver fabricated with screen printing technique at the edges of the graphene samples stripes serve as contact metals. The conductive ink with surface resistance of 5000 Ω/sq was printed on a 50 μm-thick PI film, and the thin copper foils were attached at the edges of the printed high-resistance sheet. In the fabrication process, the large-area graphene-coated PET film and the high-resistance sheet serve as the two electrodes of the graphene structure, respectively. Two pieces of 25 μm-thick polyethylene membrane soaked with an ionic liquid electrolyte are partially overlapped and sandwiched between the two electrodes. When applying the DC bias voltage on the graphene structure, the doping level of the graphene film would be controlled, resulting in the change of the surface resistance. Here, the fabricated graphene structure is used as the lossy sheet of Salisbury screen, while the high-index grating structure is made of aluminum oxide. The graphene structure is bonded with the grating structure through the glue film, and then attached to a 5 mm-thick PMI foam slab backed by a metal plate. The dimension of the whole sample is 200 mm × 200 mm.

We measured the reflectivity of the fabricated sample by using arch measurement system^30^ in the microwave anechoic chamber, as shown in Fig. 7(b). The two linearly-polarized horn antennas are placed on an arch range and the sample is located in its center. The incidence and reflection angles are both fixed at 5° to approximate the normal incidence condition. Figure 7(c) presents the measured result of the sample with the different DC biasing voltages (*V*) on the graphene layer. It is seen that when the biasing voltage is set as −1.5 V, the reflectivity of our sample is less than −10 dB between 4.4 and 18 GHz, and its fractional bandwidth reaches about 121%. This measured result agrees well with the simulated result of our structure with the graphene resistance of 500 Ω/sq. As the biasing voltage varies from −1.5 V to 3 V, the reflectivity is gradually degraded to about −8 dB, corresponding to the simulated result at the graphene resistance of 200 Ω/sq. Therefore, we have demonstrated that our sample can achieve dynamical control of the reflection over a wide frequency band.

**Figure 8:**
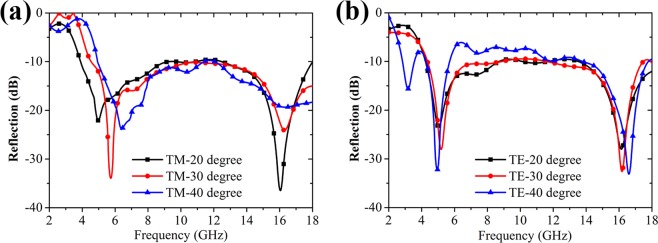
Measured reflectivity of the fabricated sample with the DC biasing voltages of −1.5 V applied on the graphene layer under different oblique incident angles. (**a**) TM polarization. (**b**) TE polarization.

In addition, we also measured the reflection characteristics of the sample applied by the DC voltage of −1.5 V under oblique incidence for both TE and TM polarizations. As Fig. 8(a) shows, as the incident angle increases from 20° to 40°, the reflection dip at the lower frequency shifts to a higher frequency and the amplitude of the reflection dip at the higher frequency gradually decreases for the TM polarizations, which is similar to the simulation result given in Fig. 5(a). For the TE polarizations, it is seen in Fig. 8(b) that the reflection amplitude degrades a little with the increase of the incident angle. However, it is noted that the reflection level of our sample still keeps about −10 dB over a relatively wide frequency band even at a large incident angle of 40°. The above measured results indicate our sample has a good angular stability.

## Discussion

In summary, the hybrid physical mechanism has been utilized to achieve broadband RCS reduction. By integrating high-index dielectric grating structure with the traditional Salisbury screen, both the absorptive loss and radiative loss are generated in our design. The high-index dielectric grating structure can produce lots of high-order reflections, and then deflect the incoming wave direction. The Salisbury screen can further absorb the incident wave energy. By tuning the surface resistance of the graphene layer that is the lossy sheet of the Salisbury screen, the RCS reduction level can be dynamically controlled. Our structure has been experimentally demonstrated to realize the 10 dB RCS reduction over a wide frequency band ranging from 4.4 to 18 GHz. In addition, the tunable RCS reduction performance can be achieved by changing the DC bias voltages applied on the graphene layer. Compared with the traditional Salisbury absorber, our approach provides a novel method for wideband and tunable RCS reduction, which may find potential application in stealth fields.
